# COVID-19-associated costs and mortality in Germany: an incidence-based analysis from a payer’s perspective

**DOI:** 10.1186/s12913-024-10838-y

**Published:** 2024-03-11

**Authors:** Florian Brandt, Giancarlo Simone, Jörg Loth, Daniel Schilling

**Affiliations:** IKK Südwest, Europaallee 3-4, Saarbrücken, 66113 Germany

**Keywords:** Cost of illness, Long COVID, Matched-pair, Population-based, Post-COVID-19 syndrome, Secondary data analysis

## Abstract

**Background:**

This study aims to estimate average COVID-19-associated healthcare costs per capita in Germany from a payer perspective. In addition, insights into COVID-19-associated mortality should be gained.

**Methods:**

For this purpose, a retrospective longitudinal analysis using health insurance claims data was performed. Patients affected by COVID-19 in Q1/2021 (investigation group (IG)) were compared to a matched non-COVID-19 control group (CG) (1:1 propensity score matching (PSM)). Mean values of healthcare costs in 2020 and 2021 were computed for both groups and then separated by age and by development of Post-COVID-19 Syndrome (PCS). Group differences were examined using Mann–Whitney U test (α = 0.05). Difference-in-Differences approach (DiD) was used to estimate average cost effects of COVID-19 in 2021. Concerning mortality, the number of deaths in 2021 was compared between IG and CG using χ^2^ test of independence.

**Results:**

A total of 8,014 insurants were included (*n* = 4,007 per group; *n* = 536 per group examining PCS patients only). Total healthcare costs varied a lot in the sample, were comparable between IG and CG in 2020, but were significantly higher in the IG in 2021 (DiD estimate = € 1,063 (in total); € 3,242 (PCS group)). This was more pronounced in the older age groups. High hospital costs of a minority of patients were the most influential driver of COVID-19-associated healthcare costs. Mortality was more than doubled in the IG (tripled in patients aged ≥ 60).

**Conclusions:**

COVID-19 is associated with significantly increased healthcare costs and mortality, especially in older age groups. The additional development of PCS further increases the costs of COVID-19.

## Background

Since its global outbreak, which started at the turn of 2019 to 2020, the coronavirus disease (COVID-19) caused enormous human, social, political, and economic costs [1]. With a view to the economic costs in Germany, economic losses of 330 Billion euros were calculated for the years 2020 and 2021 [2]. As a result of the medical treatment, that may be necessary for acute COVID-19 and its negative long-term consequences, the health system naturally also incurred enormous costs. In Germany the vast majority of medical treatment costs are not defrayed by the patients themselves but by one of nearly one hundred statutory health insurance companies. The statutory health insurance companies in turn settle reimbursement contracts with approved providers of medical care [3]. Approximately 90% of the Germans are insured within this statutory so-called third-party payer system [4]. The health services that are financed within the framework of the German statutory health insurance for insured persons are largely determined by law in terms of type, scope and price [3].

Some studies have already looked at the economic consequences of COVID-19, but largely in countries outside Europe with very different health systems [5]. There are also a few corresponding studies for the German healthcare system [5–8] and also one that took the payer perspective [6]. However, the focus here was often on an economic consideration of inpatient care [6–8]. A comprehensive study of the healthcare costs of COVID-19 in Germany from the payer perspective has not yet been carried out. Therefore, there is an urgent need for respective studies. On the one hand, they enable a much more precise assessment of the burden on the healthcare system from COVID-19 and they provide scientifically based data that can be used for cost–benefit analyses relating to the care of COVID-19 patients. In addition, they provide orientation in setting priorities within health policy – for example in the allocation of funds for research on certain diseases or in connection with the management of prevention activities [9–11]. Consequently, the primary aim of this study was to identify average healthcare costs per patient associated with COVID-19 in a population-based manner from a payer perspective. The secondary aim was to generate knowledge about COVID-19-associated mortality.

## Methods

### Study design and data base

This is a retrospective longitudinal analysis using non-public claims data of a statutory health insurance company (IKK Südwest) which insures around 640,000 people in south-west Germany, especially in Hesse (2.2%), Rhineland-Palatinate (7.6%), and the Saarland (13.6%) – the values ​​in brackets show the respective shares of the population. The available data included sociodemographic data (e. g. age and gender), medical diagnoses (according to the German Modification of the International Statistical Classification of Diseases and Related Health Problems (ICD-10-GM)), and costs of using the healthcare system (respectively reimbursements settled with the health insurance company which regularly bears the full costs within German standard care) differentiated according to healthcare areas (e. g. drug costs, hospital costs, or costs of outpatient medical care, which together represent two-thirds of the total healthcare costs within the German statutory health insurance [12]).

The effect of COVID-19 was examined in an incidence-based manner with two measurement time points over a one-year period (t0 = 2020 vs. t1 = 2021), as symptoms can last for many months [13, 14]. Insured persons who had COVID-19 in Q1/2021 (the quarter in which B.1.1.7 became the dominant variant in Germany [15] and the vaccination rates were very low, since the official vaccination start was just before that on December 27th, 2020) for the first time (so called investigation group (IG)) – defined as the presence of U07.1 (= laboratory-confirmed COVID-19) in Q1/2021 with the simultaneous absence of COVID-19-associated diagnoses (U07.1, U07.2, U08.9, U09.9, U10.9) in 2020 to avoid contamination of the results by any previous COVID-19 – were matched and compared with healthy – meaning the absence of COVID-19-associated diagnoses in 2020 and 2021 – but otherwise comparable insurants (so called control group (CG)) using propensity score matching (PSM) [16–18]. Age, gender, healthcare costs in 2020 (differentiated according to the cost areas described in the following section), and the presence respectively absence of the Top-15-Diagnoses (these are the 15 most common diagnoses made in general medical practices [19]) as an indicator of general health status were used as matching variables (nearest neighbour matching) in order to achieve a baseline that was as comparable as possible between IG and CG. The relevant diagnoses can have been made in both an outpatient and an inpatient setting. People who were not continuously insured in 2020 and 2021 were not included in this study. Of course, since mortality is an object of this study, this does not apply to deaths in 2021. The required data was used in an anonymous form. This study report is oriented towards the STROSA guidelines (Standardized Reporting Of Secondary data Analyses) [20].

### Variables of interest

The target variables considered were healthcare costs (in detail: hospital costs, costs of outpatient medical care (including psychotherapeutic care but excluding dental care), drug costs, costs of sick pay (the German statutory health insurance grants salary replacement in the event of illness), and summarized other healthcare costs (e. g. dental care, physiotherapy, and rehab)), and mortality at the end of 2021, in other words about one year after contracting COVID-19. For further clarification, the term “costs” means any costs (in Euro (€)) that are financed within the framework of the German statutory health insurance. Essentially, these are direct medical costs. In addition, productivity losses (= indirect costs) are financed by paying wage replacement benefits (these are the above mentioned “costs of sick pay”) if employees are unable to work for longer than six weeks due to illness (up to the end of six weeks there is a right to continued payment of wages from the employer).

Since the first quarter of 2021 – and therefore not an exact point in time – is the reference point for the diagnosis, the duration of COVID-19 is actually not a whole year for all persons within the IG. This is due to the fact that no exact date is recorded for diagnostic data, but only the quarter in which the diagnosis was made.

### Statistical analysis

Descriptive statistics (median, range, mean, and standard deviation (SD)) were computed for sample characteristics at t0 and for the target variables after COVID-19 at t1. The IG was differentiated into three subgroups: 1. patients with COVID-19 in Q1/2021 as previously described (IG 1, which is consistent with the entire IG), 2. patients with COVID-19 in Q1/2021 who were additionally diagnosed with a Post-COVID-19 Syndrome (PCS) diagnosis (U09.9) in the course of 2021 (IG 2), and 3. patients with COVID-19 in Q1/2021 who did not have a PCS diagnosis during 2021 (IG 3). In this way, the effect of acute COVID-19 can be differentiated from the effect of any late consequences of the illness [21, 22]. Significance of differences between the aforementioned subgroups and their respective matching partners from the CG (1:1-matching) were computed using Mann–Whitney U test, a non-parametric test for examination of group differences between two independent groups [23]. Group differences between IG2 and IG3 were examined accordingly. The Wilcoxon signed-rank test, the equivalent to the Mann–Whitney U test for dependent samples [24], was used to examine pre/post-differences within groups – meaning t0 (2020) vs. t1 (2021). To carry out group comparisons with regard to patient survival, which is a categorial variable, the χ^2^ test of independence was used instead. The significance level was set at α = 0.05 for all tests. To adjust for baseline differences between CG and IG, which are due to the lack of randomization, the Difference-in-Differences approach (DiD) – a correction of mean differences in t1 by corresponding mean differences in t0 – was used when estimating the effects [25, 26]. The results were grouped by age since an age-dependent effect of COVID-19 can be assumed [27].

The statistical analysis was performed using *Microsoft Excel*, *R (*v4.1.0 for Windows, R Foundation for Statistical Computing, 2020) combined with *R Studio* (v1.4.1717 for Windows), and *VassarStats: Website for Statistical Computation* [28].

## Results

### Sample characteristics and baseline

**Fig. 1:**
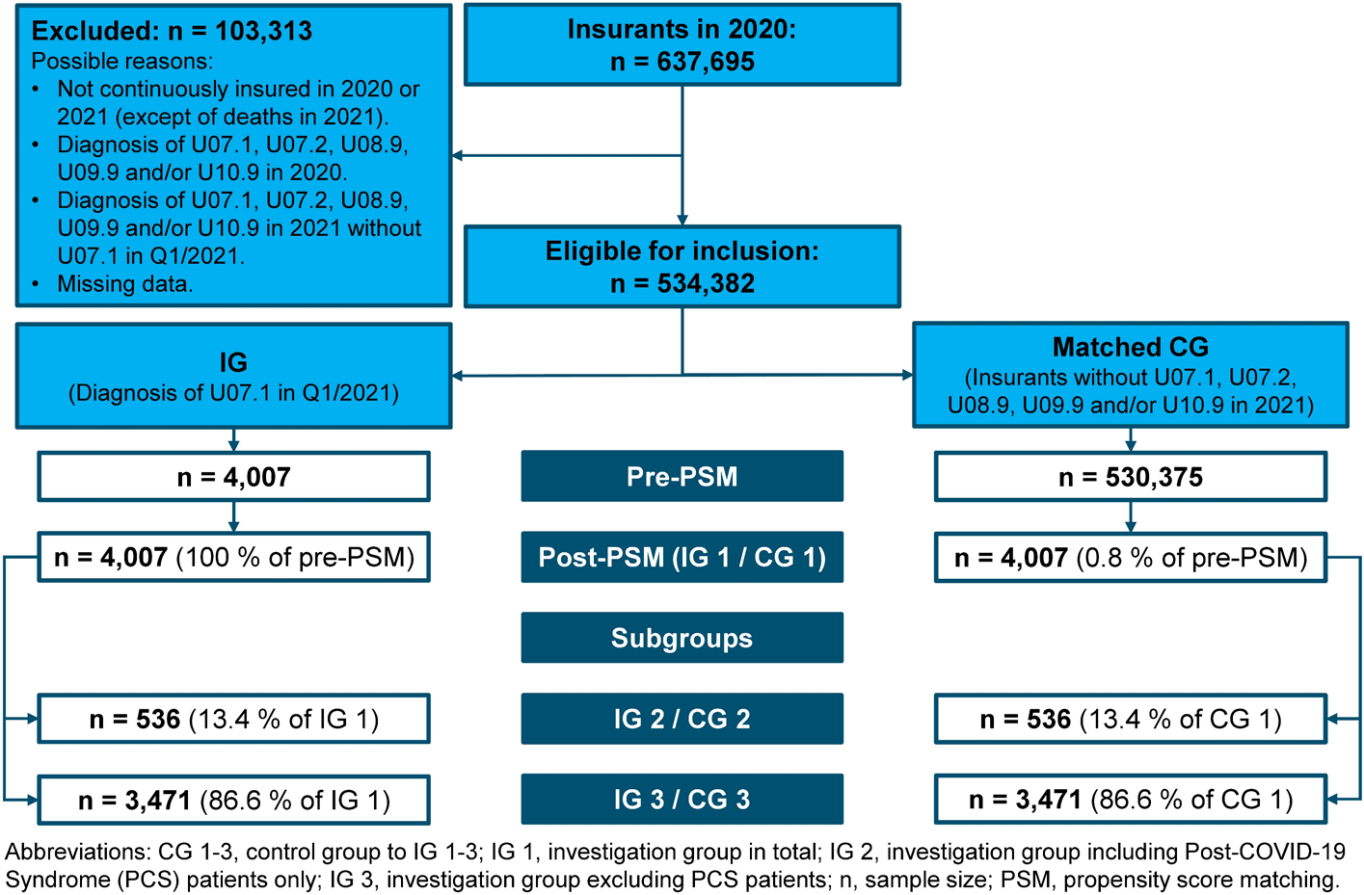
Flowchart of the inclusion process

Of the around 640,000 insurants of the IKK Südwest, 4,007 (0.6%) met the inclusion criteria of the IG and therefore were included in this study. Of these, 536 (13,4%) met the criteria for inclusion in IG 2 (COVID-19 patients who were also affected by PCS) and 3,471 (86,6%) for inclusion in IG 3 (COVID-19 patients without PCS). The same number of corresponding insurants without COVID-19 formed the CG (respectively the sub-CGs) as part of the 1:1-matching. The flowchart in Fig. 1 gives an overview of the inclusion process.

The sample characteristics respectively the baseline values of the sample (mean ± SD) are shown in Table 1: The average age in the entire sample was in the early 40 s, the gender ratio was roughly equal and the average total healthcare costs were around 3,000 €. As a result of the PSM, there were few significant differences between CG and IG at t0. Only with regard to IG 2 several significant differences were observed. This involved age, two cost ranges (outpatient medical care and drugs), and a couple of diagnoses (hypertension (I10), lipidemia (E78), back pain (M54), and depression (F32)). Furthermore, there were multiple significant differences between IG 2 and IG 3: Later PCS patients were already significantly older, more expensive and more morbid in t0 than those who did not develop PCS after COVID-19.

**Table 1:** Sample characteristics based on health insurance claims data from 2020 after PSM (shown values: *mean* ± *SD* in case of metric data respectively *n (%)* in case of categorial data; costs are shown in €)

	**CG 1** *n* = 4007	**IG 1** *n* = 4007	**CG 2** *n* = 536	**IG 2** *n* = 536	**CG 3** *n* = 3471	**IG 3** *n* = 3471
**Age (years)** ≤ 19 20–39 40–59 ≥ 60	**42.0 ± 18.8** 588 (14.7) 1096 (27.4) 1657 (41.4) 666 (16.6)	**41.8 ± 18.6** 536 (13.4) 1178 (29.4) 1680 (41.9) 613 (15.3)	**42.4 ± 17.0**^**a**^ 56 (10.4)^a^ 159 (29.7)^a^ 244 (45.5)^a^ 77 (14.4)	**46.9 ± 14.6**^**a, b**^ 28 (5.2)^a, b^ 114 (21.3)^a, b^ 318 (59.3)^a, b^ 76 (14.2)	**42.0 ± 19.1** 532 (15.3) 937 (27.0)^a^ 1413 (40.7) 589 (17.0)	**41.0 ± 19.0**^**b**^ 508 (14.6)^b^ 1064 (30.7)^a, b^ 1362 (39.2)^b^ 537 (15.5)
**Gender (f / m in %)**	**53.6 / 46.4**	**52.7 / 47.3**	**57.3 / 42.7**	**56.3 / 43.7**	**53.1 / 46.9**	**52.1 / 47.9**
**Total healthcare costs** Hospital costs Costs of outpatient med. care Drug costs Costs of sick pay Other healthcare costs	**2978 ± 8558** 927 ± 4457 622 ± 922 452 ± 2296 251 ± 1690 726 ± 4296	**3003 ± 8647** 839 ± 4248 617 ± 808 544 ± 3514 239 ± 1716 764 ± 4144	**3688 ± 9733** 1281 ± 5784 714 ± 1166^a^ 568 ± 3291^a^ 348 ± 2034 777 ± 2392	**3150 ± 6253**^**b**^ 728 ± 2405 792 ± 1008^a, b^ 487 ± 2519^a, b^ 485 ± 2675^b^ 656 ± 1782^b^	**2868 ± 8359** 872 ± 4214 608 ± 878 435 ± 2101 236 ± 1630 718 ± 4519	**2980 ± 8960**^**b**^ 856 ± 4465 590 ± 769^b^ 553 ± 3644^b^ 201 ± 1511^b^ 781 ± 4398^b^
**Top-15 Diagnoses** Essential Hypertension (I10) Lipidemia (E78) Back pain (M54) Type 2 diabetes (E11) Other non-toxic goiter (E04) Chronic ischemic heart disease (I25) Obesity (E66) Depressive episode (F32) Other diseases of the liver (K76) Gastroesophageal Reflux (K21) Gastritis and duodenitis (K29) Acute infect. in upper resp. tract (J06) Other COPD (J44) Varices of the lower extremities (I83) Bronchial asthma (J45)	982 (24.5) 666 (16.6) 1235 (30.8) 342 (8.5) 380 (9.5) 166 (4.1) 493 (12.3) 512 (12.8) 234 (5.8) 362 (9.0) 349 (8.7) 1057 (26.4) 159 (4.0) 250 (6.2) 394 (9.8)	1009 (25.2) 667 (16.6) 1238 (30.9) 322 (8.0) 338 (8.4) 194 (4.8) 510 (12.7) 497 (12.4) 237 (5.9) 357 (8.9) 325 (8.1) 1024 (25.6) 165 (4.1) 249 (6.2) 388 (9.7)	134 (25.0)^a^ 87 (16.2)^a^ 172 (32.1)^a^ 37 (6.9) 57 (10.6) 20 (3.7) 66 (12.3) 75 (14.0)^a^ 35 (6.5) 56 (10.4) 54 (10.1) 172 (32.1) 22 (4.1) 45 (8.4) 62 (11.6)	173 (32.3)^a, b^ 130 (24.3)^a, b^ 206 (38.4)^a, b^ 46 (8.6) 64 (11.9)^b^ 31 (5.8) 83 (15.5)^b^ 102 (19.0)^a, b^ 44 (8.2)^b^ 72 (13.4)^b^ 58 (10.8) 151 (28.2) 27 (5.0) 38 (7.1) 72 (13.4)^b^	848 (24.4) 579 (16.7) 1063 (30.6) 305 (8.8) 323 (9.3) 146 (4.2) 427 (12.3) 437 (12.6) 199 (5.7) 306 (8.8) 295 (8.5) 885 (25.5) 137 (3.9) 205 (5.9) 332 (9.6)	836 (24.1)^b^ 537 (15.5)^b^ 1032 (29.7)^b^ 276 (8.0) 274 (7.9)^b^ 163 (4.7) 427 (12.3)^b^ 395 (11.4)^b^ 193 (5.6)^b^ 285 (8.2)^b^ 294 (8.5) 873 (25.2) 138 (4.0) 211 (6.1) 316 (9.1)^b^

*Abbreviations*: *CG 1–3* Control group to IG 1–3, *COPD* Chronic obstructive pulmonary disease, *f* female, *IG 1* Investigation group in total, *IG 2* Investigation group including Post-COVID-19 Syndrome (PCS) patients only, *IG 3* Investigation group excluding PCS patients, *m* male, n sample size, *PSM* Propensity score matching, *SD* Standard deviation

^a^Significant difference (*p* < 0.05) between IG and respective CG (Mann–Whitney U test in case of metric data, χ^2^ independence test in case of categorial data)

^b^Significant difference (*p* < 0.05) between IG 2 and IG 3 (Mann–Whitney U test in case of metric data, χ^2^ independence test in case of categorial data)

Further measures of location and dispersion (median and range) – both for t0 and t1 – can be found in Table 2. In particular, it can be seen here that the value ranges in all cost areas were very broad and that the medians of hospital costs and costs of sick pay were zero in all groups. The median of total healthcare costs in the entire sample was around 800 € in 2020. In the IG it increased to over 1,000 € in 2021, while it stagnated in the CG.

**Table 2:** Further measures of location and dispersion – healthcare costs pre- and post-COVID-19 based on health insurance claims data from 2020 and 2021 (shown values: *median (range)* ^*a*^ in €)

	CG 1 *n =* 4007	IG 1 *n =* 4007	CG 2 *n =* 536	IG 2 *n =* 536	CG 3 *n =* 3471	IG 3 *sn =* 3471
**2020** (t0)						
**Total healthcare costs**	**806 (-4142**–**279465)**	**820 (0**–**214806)**	**961 (0–125284)**	**1029 (0–55495)**	**790 (-4142–279465)**	**779 (0–214806)**
Hospital costs	0 (-102–112939)	0 (-264–131337)	0 (0–87902)	0 (0–21345)	0 (-102–112939)	0 (-264–131337)
Costs of outpatient med. care	348 (0–16411)	360 (0–8345)	398 (0–s16411)	493 (0–8203)	340 (0–13956)	345 (0–8345)
Drug costs	36 (-46–52002)	38 (-1–130146)	40 (0–52002)	57 (0–46474)	36 (-46–42329)	35 (-1–130146)
Costs of sick pay	0 (-5589–26001)	0 (-1169–30741)	0 (0–24373)	0 (0–30741)	0 (-5589–26001)	0 (-1169–26015)
Other healthcare costs	153 (-12–238344)	167 (0–212903)	181 (0–39418)	205 (0–30676)	150 (-12–238344)	163 (0–212903)
**2021** (t1)						
**Total healthcare costs**	**811 (0**–**255982)**	**1037 (14**–**272368)**	**888 (0–85724)**	**2146 (118–111845)**	**804 (118–255982)**	**946 (14–272368)**
Hospital costs	0 (0–91893)	0 (0–233180)	0 (0–53149)	0 (0–102705)	0 (0–91893)	0 (0–233180)
Costs of outpatient med. care	348 (0–10337)	491 (0–16119)	376 (0–10337)	775 (103–10812)	345 (103–10053)	456 (0–16119)
Drug costs	36 (0–63880)	43 (0–170868)	41 (0–47751)	100 (0–17230)	35 (0–63880)	37 (0–170868)
Costs of sick pay	0 (0–30593)	0 (-677–34364)	0 (0–27693)	0 (-677–30169)	0 (-677–30593)	0 (0–34364)
Other healthcare costs	166 (0–232015)	180 (0–103439)	165 (0–40697)	334 (0–32889)	166 (0–232015)	165 (0–103439)

*Abbreviations*: *CG 1–3* Control group to IG 1–3, *IG 1* Investigation group in total, *IG 2* Investigation group including Post-COVID-19 Syndrome (PCS) patients only, *IG 3* Investigation group excluding PCS patients, *n* sample size

^a^In a few cases, the minima were negative. This is for accounting reasons. There are occasional refunds of amounts that have been paid in excess by the health insurance company. If the refund is made in a different year than the payment, there may be negative costs in the respective cost areas, namely if the refund amount is higher than the costs incurred in the billing year

### Measured effect of COVID-19

Table 3 gives an overview of the measured costs in the sample at t1. In the CG, the costs in 2021 generally did not differ significantly from those in 2020 (mean total healthcare costs in the entire sample: 3,123 €), while in the IG they tended to increase significantly (mean total healthcare costs in the entire sample: 4,211 €). In almost all age and subgroups the total healthcare costs of the IG were significantly higher than those of the CG. The situation was similar with an age-undifferentiated consideration of the individual types of costs – with a few exceptions (e. g. drug costs in CG 2 were exceptionally higher than in IG 2). In the course of an age-differentiated analysis, it is noticeable that the cost differences between IG and CG tended to be greater in the older age groups (mean total healthcare costs at 5,725 € in CG 1 patients ≥ 60 vs. 7,763 € in IG 1 patients ≥ 60). The differences between IG and CG also tended to be greater in the already more expensive PCS subgroup – with predominantly higher costs in the IG (an exception here were the higher drug costs in CG 2, which, however, were already higher at baseline). The differences between IG and CG were most pervasive when it comes to costs of outpatient medical care, where they were consistently significant across all age respectively subgroups. Furthermore, there were particularly strong mean value differences between IG and CG in hospital costs in the two oldest age groups (regarding each subgroup, e.g. 3,560 € in IG 1 patients ≥ 60 vs. 1,878 € in CG 1 patients ≥ 60) as well as in costs of sick pay in the age group from 40 to 59 (1,756 € in IG 2 vs. 537 € in CG 2). The zero medians shown in Table 2, with regard to hospital costs and costs of sick pay, have already been pointed out in the previous section.

**Table 3:** Costs after COVID-19 based on health insurance claims data from 2021 (*mean* ± *SD* in €)

	**CG 1**	**IG 1**	**CG 2**	**IG 2**	**CG 3**	**IG 3**
**Total healthcare costs** ≤ 19 20–39 40–59 ≥ 60	**3123 ± 8475**^**a**^ 1351 ± 3319^a^ 2310 ± 6998^a^ 3243 ± 9432^a^ 5725 ± 10491^a^	**4211 ± 11532**^**a, c**^ 1940 ± 7624^a, c^ 2587 ± 9594^a, c^ 4778 ± 13166^a, c^ 7763 ± 11999^a, c^	**3449 ± 8219**^**a**^ 1862 ± 4679^a^ 2482 ± 6389^a^ 3400 ± 7031^a^ 6753 ± 14208^a^	**6153 ± 10596**^**a, b, c**^ 1557 ± 1228^a, b^ 3598 ± 5570^a, b, c^ 6747 ± 11918^a, b, c^ 9194 ± 11160^a, b, c^	**3072 ± 8513**^**a**^ 1297 ± 3143^a, c^ 2280 ± 7099^a^ 3216 ± 9789^a^ 5591 ± 9911^a^	**3911 ± 11642**^**a, b, c**^ 1961 ± 7826^a, c^ 2478 ± 9925^a, c^ 4319 ± 13403^a, c^ 7560 ± 12109^a, c^
**Hospital costs** ≤ 19 20–39 40–59 ≥ 60	**907 ± 4358**^**a**^ 310 ± 1988^c^ 779 ± 4569 812 ± 4097^a^ 1878 ± 5786^a^	**1494 ± 7072**^**a, c**^ 356 ± 2244 681 ± 3705 1673 ± 9050^a, c^ 3560 ± 8071^a, c^	**947 ± 4316**^**a**^ 698 ± 3878 804 ± 4469^a^ 766 ± 3510^a^ 1994 ± 6165^a^	**2385 ± 7386**^**a, b, c**^ 382 ± 878^b^ 1244 ± 3848^a, b, c^ 2568 ± 8529^a, b, c^ 4074 ± 7248^a, c^	**901 ± 4366**^**a**^ 269 ± 1671 775 ± 4588 820 ± 4191^a^ 1863 ± 5740^a^	**1356 ± 7014**^**a, b, c**^ 354 ± 2296 620 ± 3686 1464 ± 9157^a, c^ 3487 ± 8185^a, c^
**Costs of outpatient med. care** ≤ 19 20–39 40–59 ≥ 60	**627 ± 893**^**a**^ 397 ± 726^a^ 519 ± 771^a^ 655 ± 907^a^ 938 ± 1073^a^	**757 ± 913**^**a, c**^ 506 ± 670^a, c^ 601 ± 721^a, c^ 808 ± 952^a, c^ 1135 ± 1150^a, c^	**710 ± 1017**^**a**^ 410 ± 456^a^ 596 ± 844^a^ 739 ± 1098^a^ 1075 ± 1250^a^	**1074 ± 1057**^**a, b, c**^ 641 ± 454^a, b, c^ 876 ± 730^a, b, c^ 1100 ± 968^a, b, c^ 1420 ± 1699^a, b, c^	**614 ± 872**^**a**^ 395 ± 749^a, c^ 506 ± 758^a^ 640 ± 870^a^ 920 ± 1048^a^	**708 ± 878**^**a, b, c**^ 499 ± 679^a, c^ 572 ± 714^a, c^ 740 ± 935^a, c^ 1095 ± 1046^a^
**Drug costs** ≤ 19 20–39 40–59 ≥ 60	**491 ± 2460**^**a**^ 111 ± 803^a, c^ 234 ± 1468 559 ± 2485^a^ 1080 ± 4045	**717 ± 5082**^**a, c**^ 412 ± 5001^a^ 610 ± 6530^c^ 749 ± 4420^a, c^ 1103 ± 3367	**555 ± 2905**^**a**^ 84 ± 215^a^ 172 ± 757^a^ 610 ± 2768^a^ 1515 ± 5680	**480 ± 1519**^**a, b, c**^ 129 ± 209^a, b^ 286 ± 1289^a, b, c^ 483 ± 1589^a, b, c^ 886 ± 1731	**481 ± 2384**^**a**^ 114 ± 842^a, c^ 245 ± 1557 551 ± 2433^a^ 1023 ± 3782	**754 ± 5427**^**a, b**^ 427 ± 5136^a^ 645 ± 6857 811 ± 4847^a, c^ 1133 ± 3538
**Costs of sick pay** ≤ 19 20–39 40–59 ≥ 60	**297 ± 1847**^**a**^ 1 ± 30 269 ± 1825^a^ 379 ± 1852^a^ 402 ± 2532	**397 ± 2200**^**a, c**^ 2 ± 47 204 ± 1523^a, c^ 703 ± 2943^a, c^ 278 ± 1730	**432 ± 2207**^**a**^ 0 ± 0 388 ± 2102^a^ 537 ± 2127^a^ 506 ± 3236	**1276 ± 3888**^**a, b, c**^ 39 ± 205^b^ 604 ± 2247^a, b, c^ 1756 ± 4644^a, b, c^ 729 ± 2549^b^	**276 ± 1784** 1 ± 32 248 ± 1774 351 ± 1780 388 ± 2428	**262 ± 1767**^**b, c**^ 0 ± 6 161 ± 1419^c^ 457 ± 2311^c^ 214 ± 1573
**Other healthcare costs** ≤ 19 20–39 40–59 ≥ 60	**801 ± 4341**^**a, c**^ 531 ± 1532 508 ± 1424 838 ± 6057^c^ 1428 ± 4015^a^	**846 ± 3172**^**a, c**^ 664 ± 4542 491 ± 1968 846 ± 3222^c^ 1687 ± 3303^a^	**805 ± 2503**^**a**^ 670 ± 1292 521 ± 1377 749 ± 1740^a^ 1664 ± 5325^a^	**938 ± 2073**^**a, b, c**^ 367 ± 401 587 ± 1198^b, c^ 840 ± 1354^a, b, c^ 2085 ± 4359^a^	**800 ± 4559**^**c**^ 517 ± 1556 506 ± 1433 854 ± 6520^c^ 1397 ± 3816^a^	**831 ± 3309**^**b**^ 680 ± 4664 480 ± 2034 847 ± 3518^c^ 1631 ± 3126^a^

*Abbreviations*: *CG 1–3* Control group to IG 1–3, *IG 1* Investigation group in total, *IG 2* Investigation group including Post-COVID-19 Syndrome (PCS) patients only, *IG 3* Investigation group excluding PCS patients, *n* sample size, *SD* Standard deviation

^a^Significant difference (*p* < 0.05) between IG and respective CG (Mann–Whitney U test)

^b^Significant difference (*p* < 0.05) between IG 2 and IG 3 (Mann–Whitney U test)

^c^Significant difference (*p* < 0.05) between 2020 and 2021 (Wilcoxon signed-rank test)

**Fig. 2:**
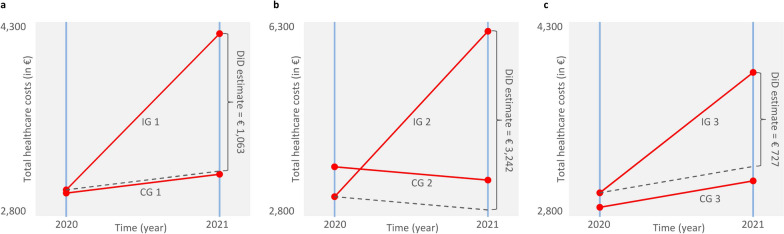
Effect of COVID-19 on total healthcare costs measured by DiD estimates **a**) in total, **b**) when only considering PCS patients, and **c**) when excluding PCS patients

The effect of COVID-19 on total healthcare costs – measured by DiD estimates – is shown in Fig. 2.

It was 1,063 euros in the total sample and more than three times as high among the PCS patients (IG 2). Table 4 shows further DiD estimates differentiated by cost areas and age groups.

**Table 4:** Effect of COVID-19 on healthcare costs measured by DiD-estimates (in €)

	**Effect of C19** in total	**Effect of C19** PCS patients only	**Effect of C19** excl. PCS patients
**Total healthcare costs** ≤ 19 years 20–39 years 40–59 years ** ≥ **60 years	**1063** 65 483 1422 2012	**3242** 655 2176 3309 5010	**727** 13 274 985 1604
**Hospital costs** ≤ 19 years 20–39 years 40–59 years ** ≥ **60 years	**675** 252 18 835 1884	**1991** 307 1020 2135 3538	**471** 235 -142 553 1656
**Costs of outpatient med. care** ≤ 19 years 20–39 years 40–59 years ** ≥ **60 years	**135** 129 153 125 131	**286** 336 304 223 357	**112** 112 136 99 100
**Drug costs** ≤ 19 years 20–39 years 40–59 years ** ≥ **60 years	**134** 4 346 54 63	**6** -35 250 -162 222	**155** 8 351 103 44
**Costs of sick pay** ≤ 19 years 20–39 years 40–59 years ** ≥ **60 years	**112** 1 16 298 -65	**707** 40 350 993 123	**21** 1 -57 124 -88
**Other healthcare costs** ≤ 19 years 20–39 years 40–59 years ** ≥ **60 years	**7** -321 17 111 -1	**254** 6 252 120 770	**-32** 341 -15 106 -108

*Abbreviations*: *C19* COVID-19, *PCS* Post-COVID-19 Syndrome

From this point of view, COVID-19 was associated with substantial additional costs overall. Here, too, it is clear that the effect tended to be stronger in the older age groups. The DiD estimates were particularly high in the PCS group (IG 2) with regard to hospital costs (1,991 € in the entire PCS group and 3,538 € among PCS patients ≥ 60), and with regard to the costs of sick pay (707 € in the entire PCS group and at 993 € particularly high in the age group from 40 to 59).

**Fig. 3:**
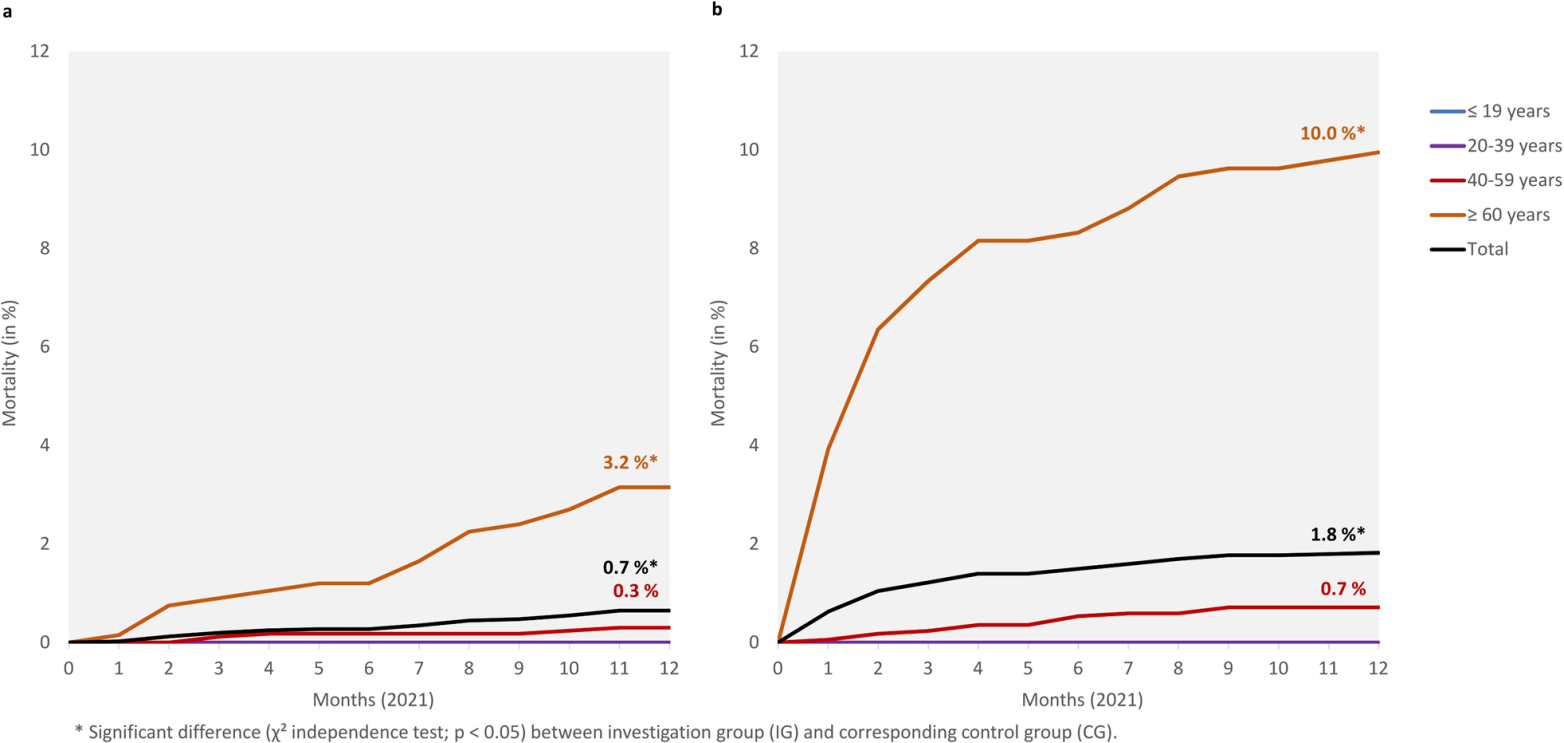
Progression of mortality in 2021 between **a**) CG and **b**) IG differentiated by age groups and overall

The progression of mortality is shown in Fig. 3.

In the two younger age groups, mortality was zero in both the IG and the CG. In the age group from 40 to 59, mortality was about twice as high and in the age group ≥ 60 about three times as high in the IG. Overall, mortality in the IG was more than doubled. In the IG, the mortality rate increased particularly sharply in the first three to four months of 2021 (that means during the period of COVID-19), while it approached the progression of the mortality rate of the CG in the further course of the year.

## Discussion

The aim of this study was to examine the effect of COVID-19 on costs and mortality in Germany from a payer perspective, especially to enable scientifically based cost–benefit analyses relating to the care of COVID-19 patients. For this purpose, claims data from 8,014 insurants of a statutory health insurance company were evaluated within a matched pair design (CG (4,007) vs. IG (4,007)). The fact that there were largely no significant differences between IG and CG at baseline speaks for a good matching result. Only IG 2 (the later PCS patients) and CG 2 differed significantly in some variables at baseline, which is presumably related to the increased morbidity of IG 2 compared to IG 3: the more specific a person's state of health becomes as a result of multimorbidity, the more difficult it becomes to find their statistical twin. The fact that 13.4% of the included COVID-19 patients developed PCS is consistent with relevant studies, which assume a PCS rate of 10–15% [29]. Also, some comorbidities already associated with the development of PCS – e. g. depression, back pain, obesity, and asthma [30] – were significantly more common in the PCS group. However, the comorbidity with the largest association measured within the previously quoted study – COPD [30] – was not significantly more frequent in the PCS group here. Furthermore, the proportion of women in the PCS group was not significantly increased here, while other studies have shown that female sex is a risk factor for the development of PCS [29, 30]. In contrast, the mean age in the PCS group was significantly higher, although older age has not been consistently associated with a higher risk of PCS development in other studies [29, 30]. Possibly, the age association here was mediated by age-associated increased COVID-19 disease severity [27], which in turn is associated with the development of PCS [31].

As expected, the healthcare costs in the IG were generally significantly higher than in the CG at t1. In view of the study situation, it is plausible that the COVID-19-associated healthcare costs tended to increase with age, since an increase in COVID-19 disease severity due to age has already been reported several times [27]. In connection with hospitalizations, a systematic review reported an increase in risk by 3.4% per age year [27]. This is in line with the results of this study, which identified hospital costs as the largest cost driver of COVID-19-associated healthcare costs – with a stronger influence in the older age groups. Since a severe course of COVID-19 is associated with a higher risk of developing PCS [31], it is not surprising that the average costs of illness were higher in the PCS subgroup. In addition, healthcare costs are of course produced by PCS itself. This was clearly recognizable here in the increased costs of sick pay in affected persons of employable age. Apparently, PCS can lead to longer periods of incapacity to work and thus to the need for salary replacement by the health insurance. With regard to outpatient medical care, there were consistently increased costs in the PCS group, too. This is also understandable, as the variety and fluctuating duration of individual PCS symptoms [29] can result in uncoordinated use of medical practices. There are some therapeutic approaches for PCS patients that are the subject of large-scale research projects in Germany. One example is the WATCH project, in which those affected complete training sessions for concentration and attention, graduated rehabilitation sports programs and behav-ioral therapy offers for a total of twelve weeks. The project also examines cost effects and is funded by the Innovation Fund of the Federal Joint Committee [32].

That the costs of COVID-19, on average, increase with age and that hospitalizations are a major cost driver was consistent across countries [5]. So far, no comparable study has been carried out in Germany that could be used to compare the specific costs of COVID-19 determined here. Cost projections for the United States have estimated direct medical costs of $ 3,045 for a COVID-19 patient [33] and $ 9,000 for a PCS patient [34]. It is true that a comparison of absolute costs does not make sense immediately, since the pricing of medical care services sometimes differs significantly between the health systems of different countries. It is interesting, however, that the cost ratio between COVID-19 patients and PCS patients roughly corresponds to the cost ratio determined here (1:3). Healthcare systems are organized nationally, which, as already mentioned, means that their financing systems and pricing mechanisms vary internationally. The international transferability of the cost results is therefore limited. Nevertheless, they can serve as a rough benchmark for other countries, especially EU states with structurally similar health systems (Note: The OECD and the European Observatory on Health Systems and Policies prepared a set of Country Health Profiles online, covering all EU Member States [35]). Against this background, it is worth mentioning that Germany has the highest health expenditure per capita among the EU member states (28% above the EU average) [36], which means that the costs measured here probably provide an upper benchmark in an international comparison. The weighting of the different types of expenditure (inpatient care, outpatient care, pharmaceutical care, etc.) is similar to the EU as a whole [36]. Since direct medical costs in Germany are almost entirely financed by the health insurance companies, they are covered very comprehensively in this study. The results on mortality can serve as a guide in countries with comparable structural characteristics and comparable access to health care services.

That COVID-19 seemed to be associated with increased mortality in older age groups only is consistent with relevant studies that identified age as one of the most important risk factors with regard to COVID-19-associated mortality [37–39]. The strong increase in mortality in the oldest age group of the IG within the first three to four months with a normalization in the following months suggests that in the observation period especially acute COVID-19 itself and not its later consequences were associated with increased mortality.

## Limitations

Although this study created comprehensive knowledge about COVID-19-asscociated healthcare costs and mortality in Germany in 2021, there were certain limitations. Due to the lack of randomization, it cannot be ruled out that the observed effects were contaminated by unknown confounding factors. Although this risk was reduced by the PSM with a broad set of matching variables carried out here, a certain risk of bias still remains [16–18]. Furthermore, the study participants were not controlled to determine whether the distribution of relevant characteristics is representative of the German population. Since only data from insured persons of the IKK Südwest, i.e. from the southwest of Germany, were analyzed, the representativeness with regard to Germany as a whole is limited. However, in comparison with Germany as a whole, there are no such differences in the federal states considered that fundamentally preclude transferability. Indicators for this are provided, for example, by the comparable overall morbidity indices [40] and cause of death statistics [41]. The age grouping enables the results to be transferred to populations with varying age structures by adjusting to their specific age structure. As stated in the background section, the health services that are financed for the study participants are largely determined by law in terms of type, scope and price. In other words: The measured costs would be – more or less – the same if the included patients were insured with another health insurance company.

The wide ranges and high standard deviations correspond to expectations: While COVID-19 is asymptomatic in many patients, others struggle with serious consequences and require hospitalization. This is reflected in the wide ranges and high standard deviations of costs, which make it clear that the results can be used to assess the impact of COVID-19 collectively, but not in individual cases. In addition, the zero medians of hospital costs and costs of sickpay in IG 1, IG 2, and IG 3 show that the majority of patients do not cause any COVID-19-associated costs with regard to the respective cost areas, but that a large proportion of the determined cost effects is due to a cost-intensive minority. Furthermore, there was probably a high number of unreported – respectively not coded – cases, especially among asymptomatic cases. The inclusion of such cases that are not coded because they do not require medical treatment would probably weaken the measured effects. This is not particularly problematic given that this study is primarily intended to provide a database for future cost–benefit analyses relating to the care of COVID-19 patients. Of course, in such analyses, the main focus is on cases that actually make use of the health system's resources.

Another limitation is that the measured values ​​refer to the year 2021. As a result of changing virus variants and also an evolving healthcare system, the findings are not necessarily transferrable to the future. Initially, PCS was not a widely recognized disease and, as a result, may have been underdiagnosed. Undiagnosed cases (i.e. cases not recorded in the underlying data) presumably have a cost effect that could not be measured here. Furthermore, vaccination rates were low in the first quarter of 2021. This limitation is also a strength in that it allowed the “raw” effect of COVID-19 to be measured. This study thus offers a primordial benchmark for all follow-up studies that examine cost or mortality effects under certain prevention measures or therapy regimes. Against this background, in future studies it would be interesting to investigate how expenditures and mortality changed as vaccination rates increased. Finally, the present study was limited to an incidence-based annual observation. Future studies could extend the observation period for further assessment of the effect of PCS or carry out a prevalence-based analysis of PCS patients.

## Conclusions

In the study population, COVID-19 was associated with average costs of € 1,063 per patient in an observation period of 9–12 months after disease onset. If only those patients who also developed PCS were considered, the corresponding amount was even € 3,242. Hospital costs were identified as key cost driver. Especially with regard to PCS patients, costs of outpatient medical care and costs of sickpay were also considerable. Overall, costs of COVID-19 increased with age. Mortality was about twice as high among the COVID-19 patients considered, and even three times as high if only patients aged 60 + were considered. A target-group-specific prevention of COVID-19, but also a more systematic PCS care, can thus substantially contribute to an improvement of the effectiveness and efficiency of German healthcare.

## Data Availability

The datasets generated and analyzed during the current study are not publicly available because they are subject to the business secrets of IKK Südwest but are available from the corresponding author on reasonable request.

## Abbreviations

CG: Control group
COVID-19: Coronavirus disease
DiD: Difference in differences
€: Euro
ICD-10-GM: German Modification of the International Statistical Classification of Diseases and Related Health Problems
IG: Investigation group
n: Sample size
PCS: Post-COVID-19 Syndrome
PSM: Propensity score matching
t0: Year 2020
t1: Year 2021

## References

[1] Bonotti M, Zech ST, Bonotti M, Zech ST (2021). The Human, Economic, Social, and Political Costs of COVID-19. Recovering Civility during COVID-19.

[2] ifo Institute. Coronavirus Pandemic Caused EUR 330 Billion in Economic Losses for Germany. Munich; 2022. Available at: https://www.ifo.de/en/press-release/2022-02-17/coronavirus-pandemic-caused-eur-330-billion-economic-losses-germany. Accessed 02 Feb 2023.

[3] Busse R, Schreyögg J, editors. Management im Gesundheitswesen. 5th ed. Berlin/Heidelberg: Springer; 2022. p. 1–10. 10.1007/978-3-662-64176-7.

[4] GKV Spitzenverband [Central Federal Association of the Statutory Health Insurance Companies]. Zahlen und Grafiken – Kennzahlen der gesetzlichen Krankenversicherung – Versicherte je System in Prozent [Figures and graphics – key figures of the statutory health insurance – insured per system in percent]. Berlin; 2022. Available at: https://www.gkv-spitzenverband.de/service/zahlen_und_grafiken/zahlen_und_grafiken.jsp. Accessed 03 Feb 2023.

[5] Richards F, Kodjamanova K, Chen X (2022). Economic Burden of COVID-19: A Systematic Review. Clinicoecon Outcomes Res.

[6] Jeck J, Jakobs F, Kron A (2022). A cost of illness study of COVID‑19 patients and retrospective modelling of potential cost savings when administering remdesivir during the pandemic “frst wave” in a German tertiary care hospital. Infection.

[7] Gandjour A (2021). How many intensive care beds are justifiable for hospital pandemic preparedness? A cost-effectiveness analysis for COVID-19 in Germany. Appl Health Econ Health Policy.

[8] Karagiannidis C, Mostert C, Hentschker C (2020). Case characteristics, resource use, and outcomes of 10 021 patients with COVID-19 admitted to 920 German hospitals: an observational study. Lancet Respir Med.

[9] Shiell A, Gerard K, Donaldson C (1987). Cost of illness studies: An aid to decision-making?. Health Policy.

[10] Bloom BS, Bruno DJ, Maman DY (2001). Usefulness of US cost-of-illness studies in healthcare decision making. Pharmacoeconomics.

[11] Shaya FT, Mullins CD, Wong W (2002). Incidence versus prevalence modeling in pharmacoeconomics. Expert Rev Pharmacoeconomics Outcomes Res.

[12] GKV Spitzenverband [Central Federal Association of the Statutory Health Insurance Companies]. GKV-Kennzahlen – Ausgaben für einzelne Leistungsbereiche der GKV 2021 in Prozent [GKV key figures – expenditure for individual service areas of the GKV 2021 in percent]. Berlin; 2022. Available at: https://www.gkv-spitzenverband.de/service/zahlen_und_grafiken/gkv_kennzahlen/gkv_kennzahlen.jsp. Accessed 03 Feb 2023.

[13] Tran V, Porcher R, Pane I, Ravaud P (2022). Course of post COVID-19 disease symptoms over time in the ComPaRe long COVID prospective e-cohort. Nat Commun.

[14] Lopez-Leon S, Wegman-Ostrosky T, Perelman C (2021). More than 50 long-term effects of COVID-19: a systematic review and meta-analysis. Sci Rep.

[15] Robert Koch Institute (RKI). Bericht zu Virusvarianten von SARS-CoV-2 in Deutschland [Report on virus variants of SARS-CoV-2 in Germany]. Berlin; 12 Mai 2021. Available at: https://www.rki.de/DE/Content/InfAZ/N/Neuartiges_Coronavirus/DESH/Bericht_VOC_2021-05-12.pdf?__blob=publicationFile. Accessed 03 Feb 2023.

[16] Rosenbaum PR, Rubin DB (1983). The central role of the propensity score in observational studies for causal effects. Biometrika.

[17] D’Agostino RB (1998). Propensity score methods for bias reduction in the comparison of a treatment to a non-randomized control group. Stat Med.

[18] Austin PC (2011). An introduction to propensity score methods for reducing the effects of confounding in observational studies. Multivar Behav Res.

[19] Gesundheitsberichterstattung des Bundes [Federal health reporting]. Häufigste Diagnosen in Prozent der Behandlungsfälle in Arztpraxen in Nordrhein (Rang und Anteil). Gliederungsmerkmale: Jahre, Nordrhein, Geschlecht, ICD10, Arztgruppe [Most common diagnoses as a percentage of treatment cases in medical practices in North Rhine (rank and share). Structural features: years, North Rhine-Westphalia, gender, ICD10, physician group]. Berlin; 27 July 2016. Available at: https://www.gbe-bund.de/gbe/pkg_isgbe5.prc_menu_olap?p_uid=gast&p_aid=22070480&p_sprache=D&p_help=0&p_indnr=638&p_indsp=&p_ityp=H&p_fid=. Accessed 03 Feb 2023.

[20] STandardisierte BerichtsROutine für Sekundärdaten Analysen (STROSA) – ein konsentierter Berichtsstandard für Deutschland, Version 2 [A Consensus German Reporting Standard for Secondary Data Analyses, Version 2 (STROSA-STandardisierte BerichtsROutine für SekundärdatenAnalysen)]. Gesundheitswesen. 2016;78(Suppl. 1):e145–e160. 10.1055/s-0042-108647 10.1055/s-0042-112008 27428525

[21] Oronsky B, Larson C, Hammond TC (2023). A Review of Persistent Post-COVID Syndrome (PPCS). Clin Rev Allergy Immunol.

[22] Anaya J-M, Rojas M, Salinas ML (2021). Post-COVID syndrome. A case series and comprehensive review. Autoimmun Rev.

[23] Mann HB, Whitney DR (1947). On a test of whether one of two random variables is stochastically larger than the other. Ann Math Statist.

[24] Wilcoxon F (1945). Individual comparisons by ranking methods. Biometrics Bulletin.

[25] Gertler PJ, Martinez S, Premand P (2016). Impact Evaluation in Practice.

[26] Wing C, Simon K, Bello-Gomez RA (2018). Designing Difference in Difference Studies: Best Practices for Public Health Policy Research. Annu Rev Public Health.

[27] Starke K, Reissig D, Petereit-Haack G (2021). The isolated effect of age on the risk of COVID-19 severe outcomes: a systematic review with meta-analysis. BMJ Global Health.

[28] VassarStats: Website for Statistical Computation. Available at: http://vassarstats.net/. Accessed 06 Feb 2023.

[29] Nalbandian A, Desai AD, Wan EY (2023). Post-COVID-19 Condition. Annu Rev Med.

[30] Subramanian A, Nirantharakumar K, Hughes S (2022). Symptoms and risk factors for long COVID in non-hospitalized adults. Nat Med.

[31] Kamal M, Abo Omirah M, Hussein A, Saeed H (2021). Assessment and characterisation of post-COVID-19 manifestations. Int J Clin Pract.

[32] Gemeinsamer Bundesausschuss [Federal Joint Committee]. WATCH – Mobile wohnortnahe Versorgung zur Steuerung der sektorübergreifenden Therapie bei Post-COVID-19 in Thüringen [WATCH – Mobile care close to home to control cross-sector therapy for post-COVID-19 in Thuringia]. Available at: https://innovationsfonds.g-ba.de/projekte/neue-versorgungsformen/watch-mobile-wohnortnahe-versorgung-zur-steuerung-der-sektoruebergreifenden-therapie-bei-post-covid-19-in-thueringen.574. Accessed 05 Oct 2023.

[33] Bartsch SM, Ferguson MC, McKinnell JA (2020). The Potential Health Care Costs And Resource Use Associated With COVID-19 In The United States. Health Aff (Millwood).

[34] Cutler DM (2022). The Costs of Long COVID. JAMA Health Forum.

[35] OECD & European Observatory on Health Systems and Policies. Country Health Profiles. Available at: https://health.ec.europa.eu/state-health-eu/country-health-profiles_en. Accessed 05 Oct 2023.

[36] OECD & European Observatory on Health Systems and Policies (2021). Germany: Country Health Profile 2021, State of Health in the EU.

[37] Mehraeen E, Karimi A, Barzegary A (2020). Predictors of mortality in patients with COVID-19–a systematic review. Eur J Integr Med.

[38] Kang SJ, Jung SI (2020). Age-Related Morbidity and Mortality among Patients with COVID-19. Infect Chemother.

[39] Shi C, Wang L, Ye J (2021). Predictors of mortality in patients with coronavirus disease 2019: a systematic review and meta-analysis. BMC Infect Dis.

[40] Gesundheitsberichterstattung des Bundes [Federal health reporting]. Todesursachenstatistik [Cause of death statistics]. Available at: https://www.gbe-bund.de/gbe/pkg_isgbe5.prc_menu_olap?p_uid=gast&p_aid=92356495&p_sprache=D&p_help=0&p_indnr=6&p_indsp=658&p_ityp=H&p_fid=. Accessed 05 Oct 2023.

[41] BARMER Institut für Gesundheitssystemforschung (bifg) [BARMER Institute for Health Systems Research]. Morbiditäts- und Sozialatlas [Morbidity and Social Atlas]. Available at: https://www.bifg.de/atlas. Accessed 05 Oct. 2023.

